# Bipolar Affective Disorder and Migraine

**DOI:** 10.1155/2012/389851

**Published:** 2012-05-09

**Authors:** Birk Engmann

**Affiliations:** Department of Neurology, Fachklinikum Brandis, Am Wald, 04821 Brandis, Germany

## Abstract

This paper consists of a case history and an overview of the relationship, aetiology, and treatment of comorbid bipolar disorder migraine patients. A MEDLINE literature search was used. Terms for the search were bipolar disorder bipolar depression, mania, migraine, mood stabilizer. Bipolar disorder and migraine cooccur at a relatively high rate. Bipolar II patients seem to have a higher risk of comorbid migraine than bipolar I patients have. The literature on the common roots of migraine and bipolar disorder, including both genetic and neuropathological approaches, is broadly discussed. Moreover, bipolar disorder and migraine are often combined with a variety of other affective disorders, and, furthermore, behavioural factors also play a role in the origin and course of the diseases. Approach to treatment options is also difficult. Several papers point out possible remedies, for example, valproate, topiramate, which acts on both diseases, but no first-choice treatments have been agreed upon yet.

## 1. Introduction

Both bipolar disorder and migraine are not uncommon diseases. Whereas disorders of bipolar spectrum have an occurrence rate between 0.4 and 1.4% depending on different studies [1], migraine has rates from 10 to 30% [22]. More often than noticed, bipolar disorder occurs together with migraine. In a study of patients suffering from bipolar disorder (both type I and II), Ortiz et al. [2] found comorbid migraine in 24.5% of all bipolar cases and McIntyre et al. [3] of 24.8% (versus a general population rate of 10.3%). Contrary to that, Holland et al. [4] found a much lower prevalence of 4.7%. Nevertheless, comorbidity of both diseases seems to be an underestimated problem. In short, the main characteristics of bipolar disorder are recurrent episodes of depressive and manic states. Migraine is a usually unilateral headache often combined with nausea, photophobia, and others. As with bipolar disorder, migraine is divided into different subtypes, such as migraine with aura and without, or familial hemiplegic migraine among others.

## 2. Case History

A patient in his middle fifties has been suffering from mood disturbances since adolescence. The patient's family history hints of “nerve diseases” of his mother and of his aunt and grandmother on the mother's side. For several days, he was overactive and, as his brother once told him, “mad.” The patient, during his hyperactive phase, often tried to convince his brother to do wayward things. After some days of hyperactivity, the patient's mood switched overnight to being deeply depressed, with avolition and a gloomy mood that lasted for many weeks. When this pattern first appeared, it occurred every one or two months; but, in its later appearances, the switching occurred monthly or within a fortnight. The patient never underwent therapy. At the age of 20, he had a head injury caused by an accident with his motorcycle. (More specific data about this event were not available.) After that, a migraine developed, which at first was never severe, and occurred four times a year. These early migraines occurred independently of his bipolar disorder. In the last ten years, however, the migraine became extremely severe on occasion and was linked to his bipolar disorder. The linkage is that manic states were always followed by migraine attacks with depressive mood. With these severe migraines, the patient experiences withdrawal, loss of interests, sleep disturbances, and recurrent suicidal fantasies. In addition to these migraines which follow after manic states, other migraines occur independently of manic states. In these last ten years, severe migraine attacks begin with aura symptoms such as a zigzag pattern in visual field, narrowing of visual field (tunnel view), blurred vision, and flashes. Then, speech arrest follows and palsy develops starting with lips, face, neck, and extremities, mostly on the right site. Anterograde amnesia follows on such a phase which lasts some hours in different degrees of severity. Speech arrest is usually the longest lasting symptom. Migraine headaches endure from 1 to 3 days. Manic states usually last a few days, depressive ones some weeks.

In recent, MRI scan was nothing abnormal detected. EEG in symptom-free intervals revealed an unstable alpha rhythm but no major changes. Routine laboratory data and parameters for thyroid gland, borreliosis, and lues showed no abnormalities.

In the case just described, the beginning of the bipolar disorder, presumably of type I, precedes the onset of migraine attacks. It is a migraine of a complicated type. In the differential, a familial hemiplegic migraine is possible.

## 3. Relationship between Migraine and Bipolar Disorder

The literature broadly supports comorbidity of migraine and bipolar disorder. Datta and Kumar [5] reported on a 19-year-old patient with hypomania as an aura of migraine. In a review, Antonaci et al. [6] unravelled a coincidence of migraine and affective anxiety disorders and, especially, a “trend towards an association of migraine and bipolar disorder” (see also [7]). Most studies support that migraine is associated not only with bipolar disorder but also with major depression, panic disorder, social phobia [8, 9], drug abuse [10], suicide, and neurological and internal diseases too, for instance stroke or hypertension [11]. Vice versa, according to a review of literature of McIntyre et al. [12], subjects with other neurological diseases, such as epilepsy or multiple sclerosis, seem to have a higher occurrence of bipolar disorder.

**Are There Differences in Prevalence of Comorbidities?**

Dilsaver et al. [13] found a higher prevalence of migraine in bipolar patients than in those who suffer from major depressive disorder. In a study by Ortiz and colleagues [2], which distinguished bipolar I from bipolar II patients, a higher prevalence of migraine in bipolar II subjects was apparent and, in addition, higher rates were found for suicidal tendencies and anxiety disorders. Patients with a major depressive episode who had a family history of bipolar disorder had an increased risk of suffering from migraine [14]. Patients with unipolar depression and migraine have more depressive episodes in the course of time and more often a positive family history of migraine compared to those without comorbid migraine [15].

**Does Migraine Influence the Course of Bipolar Disorder?**

According to Brietzke et al. [16], patients with both bipolar I and bipolar II disorder who suffer from comorbid migraine had more mood episodes, especially depressive ones. Furthermore, they found a higher occurrence rate of other psychiatric and general medical comorbidities.

## 4. Is There a Common Aetiology of Bipolar Disorder and Migraine?

Different gene regions are suspect as being responsible for association of bipolar disorder with migraine, but results in studies differ. One recent study [17] found no evidence that polymorphisms of the genes ANK3 and CACNA1C in migraine patients were associated with an elevated risk for bipolar disorder. Another recent study [18, 19], which investigated genomewide associations, found some gene regions which differed in bipolar patients depending on whether they were susceptible to migraine or not. Holland and Agius [20] saw “much overlap in neuropathological mechanisms” in both diseases. Besides genetics, they discussed altered expression of neurotrophic factors, cellular calcium physiology, endoplasmatic reticulum function, neuronal loss and pointed out the important question of sensitisation which is still controversially discussed: do recurrent episodes in either the diseases facilitate further ones?

Others bring up white matter hyperintensities for discussion. Gunde et al. [21] argued that high rates of such signs, detected in neuroimaging, play an important role both in bipolar disorder and migraine, and a substantial proportion of the hyperintensities would be related to the comorbidity of both diseases.

## 5. Treatment Options for Comorbid Bipolar/Migraine Patients

Although there are no evidence-based treatment suggestions for people with bipolar disorder and comorbid migraine, it seems reasonable that substances which act on both bipolar disorder and migraine could be used to ward off relapse. A problem in this approach is that the particular substances which are the drugs of choice for prophylactic treatment of migraine—the beta antagonists—could make depressive symptoms worse. On the other hand, valproate, which theoretically acts on both disorders, has only an off-label status in migraine prophylaxis [22]. Nevertheless, several papers see benefits from valproate and other substances. Finocchi et al. [23] suggested valproate and topiramate as a mood stabilizer in migraine without aura and lamotrigine in migraine with aura. Even amitriptyline could be used for prevention of mood episodes and migraine attacks. Serotonin reuptake inhibitors (SSRIs) and serotonin norepinephrine reuptake inhibitors (SNRIs) are recommended in migraine patients for comorbid depression and anxiety. Caution should be exercised when triptans are combined with SSRIs or SNRIs because of a possible risk of serotonin syndrome [24].

In this vein, also neuroleptics which have already been used broadly as mood stabilizers are worth consideration, but, in relation to migraine, no sufficient studies exist [25]. Last but not least, the literature (see [26]) suggests that behavioural and cognitive-behavioural therapies are helpful for patients with a comorbid bipolar/migraine diagnosis.

## 6. Discussion

Migraine is an important comorbid disease in bipolar patients. It not only strengthens the cause of bipolar disorder but also worsens the recurrence rate with regard to depressive episodes. Bipolar II patients have a higher susceptibility of having comorbid migraine.

The literature survey with regard to the origins of both diseases indicates a variety of theories and working points. It is important to consider both the multifactor genesis of affective diseases and the influence of psychic disorders on neurological disorders such as migraine. We do not know yet whether bipolar disorder has causal or merely accidental connections with migraine. Nor do we know what organic or psychic conditions might exactly link the diseases, or why some patients have the comorbidities and others do not. Furthermore, many other psychic and neurological disorders can be comorbid with bipolar disorder.

There is no proven treatment regimen for migraine with comorbid bipolar disorder, so that pharmacological therapy is still a matter of trial and error. Nevertheless, some remedies seem to have effects on both of the diseases, but one must be wary of purchasing the benefit of a remedy in one disease by worsening the other.

## References

[1] Goodwin FK, Jamison KR (2007). *Manic-Depressive Illness*.

[2] Ortiz A, Cervantes P, Zlotnik G (2010). Cross-prevalence of migraine and bipolar disorder. *Bipolar Disorders*.

[3] McIntyre RS, Konarski JZ, Wilkins K, Bouffard B, Soczynska JK, Kennedy SH (2006). The prevalence and impact of migraine headache in bipolar disorder: results from the Canadian Community Health Survey. *Headache*.

[4] Holland J, Agius M, Zaman R (2011). Prevalence of co-morbid bipolar disorder and migraine in a regional hospital psychiatric outpatient department. *Psychiatria Danubina*.

[5] Datta S, Kumar S (2006). Hypomania as an aura in migraine. *Neurology India*.

[6] Antonaci F, Nappi G, Galli F, Manzoni GC, Calabresi P, Costa A (2011). Migraine and psychiatric comorbidity: a review of clinical findings. *Journal of Headache and Pain*.

[7] Jerrell JM, McIntyre RS, Tripathi A (2010). A cohort study of the prevalence and impact of comorbid medical conditions in pediatric bipolar disorder. *Journal of Clinical Psychiatry*.

[8] Jette N, Patten S, Williams J, Becker W, Wiebe S (2008). Comorbidity of migraine and psychiatric disorders—a national population-based study. *Headache*.

[9] Ratcliffe GE, Enns MW, Jacobi F, Belik SL, Sareen J (2009). The relationship between migraine and mental disorders in a population-based sample. *General Hospital Psychiatry*.

[10] Pompili M, Di Cosimo D, Innamorati M, Lester D, Tatarelli R, Martelletti P (2009). Psychiatric comorbidity in patients with chronic daily headache and migraine: a selective overview including personality traits and suicide risk. *Journal of Headache and Pain*.

[11] Wang SJ, Chen PK, Fuh JL (2010). Comorbidities of migraine. *Frontiers in Neurology*.

[12] McIntyre RS, Soczynska JK, Beyer JL (2007). Medical comorbidity in bipolar disorder: reprioritizing unmet needs. *Current Opinion in Psychiatry*.

[13] Dilsaver SC, Benazzi F, Oedegaard KJ, Fasmer OB, Akiskal KK, Akiskal HS (2009). Migraine headache in affectively Ill Latino adults of Mexican American origin is associated with bipolarity. *Primary Care Companion to the Journal of Clinical Psychiatry*.

[14] Dilsaver SC, Benazzi F, Oedegaard KJ, Fasmer OB, Akiskal HS (2009). Is a family history of bipolar disorder a risk factor for migraine among affectively ill patients?. *Psychopathology*.

[15] Oedegaard KJ, Fasmer OB (2005). Is migraine in unipolar depressed patients a bipolar spectrum trait?. *Journal of Affective Disorders*.

[16] Brietzke E, Moreira CL, Duarte SV Impact of comorbid migraine on the clinical course of bipolar disorder.

[17] Wöber-Bingöl Ç, Tropeano M, Karwautz A (2011). No association between bipolar disorder risk polymorphisms in ANK3 and CACNA1C and common migraine. *Headache*.

[18] Oedegaard KJ, Greenwood TA, Johansson S (2010). A genome-wide association study of bipolar disorder and comorbid migraine. *Genes, Brain and Behavior*.

[19] Oedegaard KJ, Greenwood TA, Lunde A (2010). A genome-wide linkage study of bipolar disorder and co-morbid migraine: replication of migraine linkage on chromosome 4q24, and suggestion of an overlapping susceptibility region for both disorders on chromosome 20p11. *Journal of Affective Disorders*.

[20] Holland J, Agius M (2011). Neurobiology of bipolar disorder—lessons from migraine disorders. *Psychiatria Danubina*.

[21] Gunde E, Blagdon R, Hajek T (2011). White matter hyperintensities: from medical comorbidities to bipolar disorders and back. *Annals of Medicine*.

[22] Hufschmidt A, Lücking CH (2003). *Neurologie Compact. Leitlinien für Klinik und Praxis*.

[23] Finocchi C, Villani V, Casucci G (2010). Therapeutic strategies in migraine patients with mood and anxiety disorders: clinical evidence.. *Neurological Sciences*.

[24] Evans RW, Tepper SJ, Shapiro RE, Sun-Edelstein C, Tietjen GE (2010). The FDA alert on serotonin syndrome with use of triptans combined with selective serotonin reuptake inhibitors or selective serotonin-norepinephrine reuptake inhibitors: American headache society position paper. *Headache*.

[25] Dusitanond P, Young WB (2009). Neuroleptics and migraine. *Central Nervous System Agents in Medicinal Chemistry*.

[26] Baskin SM, Smitherman TA (2009). Migraine and psychiatric disorders: comorbidities, mechanisms, and clinical applications. *Neurological Sciences*.

